# Research Progress in High-Temperature-Resistant Electromagnetic Wire

**DOI:** 10.3390/ma17102258

**Published:** 2024-05-10

**Authors:** Haomin Li, Minglu Feng, Lianning Guo, Yingsan Geng

**Affiliations:** State Key Laboratory of Electrical Insulation and Power Equipment, Xi’an Jiaotong University, Xi’an 710049, China

**Keywords:** high-temperature-resistant electromagnetic wire

## Abstract

Electromagnetic wire is the carrier of energy and signal transmission. With the rapid development in aerospace, atomic energy, and other industrial fields, there is an increasing demand for the high-temperature-resistance of electromagnetic wires. In using traditional electromagnetic wires, it is difficult to meet the current temperature-resistance requirements. Therefore, the development of high-temperature-resistant electromagnetic wire has extremely important application value. In this paper, high-temperature-resistant electromagnetic wires are divided into organic insulated high-temperature-resistant electromagnetic wires, organic–inorganic insulated composite high-temperature-resistant electromagnetic wires, and inorganic insulated high-temperature-resistant electromagnetic wires. The method of improving the temperature-resistance level of organic insulated high-temperature-resistant electromagnetic wire is introduced. The selection principle of organic–inorganic and inorganic insulation high-temperature-resistant electromagnetic-wire conductor materials is analyzed. The current research status of organic–inorganic and inorganic insulated high-temperature-resistant electromagnetic wires is reviewed. The technical routes for preparing inorganic insulated high-temperature-resistant electromagnetic wire are compared. Finally, the challenges faced by the current high-temperature-resistant electromagnetic wire are pointed out, and the future development direction of organic–inorganic-composite insulation and inorganic insulation high-temperature-resistant electromagnetic wire is proposed.

## 1. Introduction

Electromagnetic wire is a kind of conductive metal with an insulating layer, which is used to realize electromagnetic energy conversion and signal transmission. It is the heart of industrial products such as power equipment, electrical appliances, and motors in various fields. In early power-transmission and industrial applications, electromagnetic wires mainly used organic insulating materials such as rubber, plastic, and polyimide. Organic insulating materials demonstrate good performance in a specific temperature range, but in the face of the demand for extreme high-temperature environments for electrical equipment, they have the limitations of poor temperature-resistance and thermal conductivity. Although nanocomposites have been proposed to improve the high-temperature-resistance of organic insulating materials [1,2,3], they can only improve the heat-resistance level by tens of degrees Celsius, which is still not enough to meet the challenge of higher temperatures. The insulation material of the current common electromagnetic wire cannot withstand high temperatures above 300 °C, which limits the electromagnetic wire in working in extremely high-temperature environments such as those of aerospace and nuclear power.

In order to cope with the challenges of extreme environments, organic–inorganic-composite and pure inorganic insulation high-temperature-resistant electromagnetic wire came into being. High-temperature-resistant resin + ceramic/glass filler or pure ceramic and glass are used as insulation-layer materials [4]. The emergence of these two types of high-temperature-resistant-insulation electromagnetic wire marks a new exploration of electromagnetic wire insulation materials in extreme environments. The United States is the first to develop an inorganic insulated electromagnetic wire coated with nickel-plated copper wire in an extreme temperature range (−267~600 °C), and the DC is able to withstand a voltage level of 200 V. After that, Japan Sumitomo Electric Industries developed a new type of high-temperature aluminum conductor by using copper alloy and strengthened alloy with good heat resistance as a conductor material and alumina as an insulation layer [5]. The Phelps Dodge company in the United States has prepared single-layer, double-layer, and three-layer high-temperature insulated electromagnetic wires, all of which have been applied [6]. Japan’s Tokyo Jumonji et al. covered a ceramic layer on the surface of the conductor material, and then covered the surface of the ceramic layer with an organic coating to prepare a ceramic insulated electromagnetic wire [5].

At present, the preparation methods of high-temperature-resistant electromagnetic wire with pure inorganic material as an insulating layer include the slurry-coating sintering method, the sol-gel method, and the plasma electrolytic oxidation method [4,7,8]. The slurry-coating-sintering method and sol-gel method have the problems of poor wettability between the slurry, sol, and substrate during the preparation process, many film defects after film formation, and poor adhesion between the film and substrate. Although the film prepared by the plasma electrolytic oxidation method is metallurgically bonded to the substrate, it cannot be coated on valve metals such as copper. And the biggest disadvantage of a pure inorganic insulation layer is the contradiction between the brittleness of the inorganic insulation material and the inherent demand of electromagnetic wire for winding performance. The preparation of organic–inorganic combined high-temperature-resistant electromagnetic wire is to apply organic–inorganic insulation coating on the wire substrate by dipping and pulling. Although the toughness of organic materials can be used to meet the winding performance of electromagnetic wires, when the organic–inorganic composites are exposed to high temperatures, the decomposition of the organic components will lead to a large number of defects such as holes and cracks in the insulating layer, which are extremely unfavorable for the dielectric insulation performance of the insulating layer. Therefore, the current insulation-material design and preparation methods cannot simultaneously ensure the dielectric insulation performance of the high-temperature-resistant coating, the good adhesion between the insulation layer and the substrate, and the winding performance of the coating.

In this paper, the development status of high-temperature-resistant electromagnetic wire is reviewed, and the development process and current usage of organic insulated high-temperature-resistant electromagnetic wire are introduced. The selection of conductor materials, insulation materials, preparation methods, and existing products of inorganic insulated high-temperature-resistant electromagnetic wire are discussed. Finally, the current difficulties and future development trends of high-temperature-resistant electromagnetic wire are discussed.

## 2. Organic Insulated High-Temperature-Resistant Electromagnetic Wire

**Figure 1:**
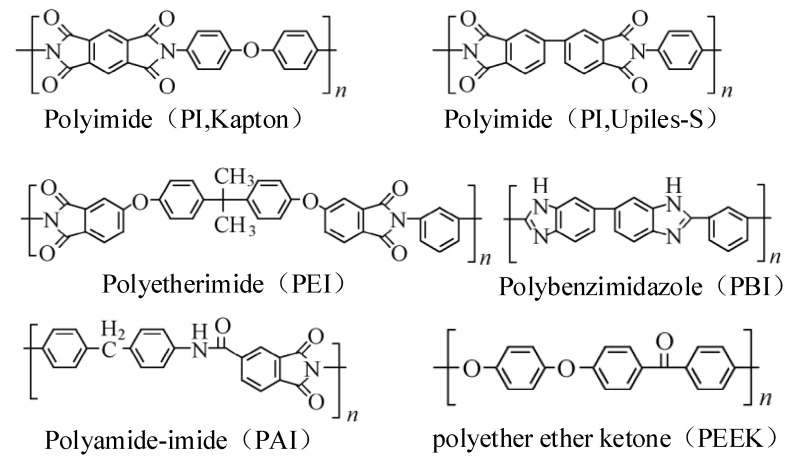
Typical chemical structure of high-temperature-resistant polymer dielectric materials.

Insulating polymer materials are one of the key development directions of modern high-temperature electrical-insulation products. Its molecular structure characteristics are obvious. There are often strong conjugate effects or chemical bonds such as hydrogen bonds, which are easily formed inside, resulting in strong interaction forces within and between molecular chains. This feature makes these materials exhibit many excellent physical and chemical properties, such as high-temperature-resistance (a high glass-transition temperature, a high thermal-decomposition temperature, and excellent high-temperature dimensional stability), excellent dielectric properties (including high insulation strength and high volume and surface resistivity), excellent environmental stability (resistance to chemical, atmospheric, and extreme space environments), and good mechanical properties [9,10,11,12]. Compared with traditional dielectric materials, the processing of polymer materials is more difficult, and the requirements for their manufacturing process are more stringent. Common insulating polymer materials include polyimide (PI), polyetheretherketone (PEEK), polytetrafluoroethylene (PTFE), polyethersulfone ether ketone (PESK), and silicone rubber, as shown in Figure 1. These insulating polymer materials are widely used in aerospace, microelectronics manufacturing, automobiles, medical equipment, and other fields due to their excellent temperature resistance, excellent mechanical properties, and excellent electrical-insulation properties [13,14,15,16].

### 2.1. Polyimide (PI) Polymer Materials

Polyimides (PIs), polyamide imides (PAIs), polyetherimides (PEIs), and polyesterimides (PEsIs) are important high-performance polymer materials, which are characterized by the presence of imide rings. This category of materials is distinguished according to the unique functional groups in their molecules: PI is the basic form of imide materials, while PAI is characterized by the introduction of amide bonds, PEI combines ether bonds, and PEsI is distinguished by the presence of ester bonds. Polyimide (PI) has excellent thermal stability, extreme temperature resistance (its initial thermal decomposition temperature generally exceeds 500 °C, and it will not crack in liquid helium at −269 °C), excellent mechanical strength, good flame-retardant properties, and excellent electrical-insulation properties [17]. As a new type of polymer material, polyamide imide (PAI) not only inherits the high-temperature resistance of polyimide (PI), but also combines the high-strength characteristics of polyamide, showing an excellent comprehensive performance. In the electrical industry, PAI is mainly made into insulating paints and plastics, and is widely used in the insulation layer of copper wires. In addition to the imide ring, polyetherimide (PEI) polymer materials also have substitution groups such as ether bonds and isopropyl groups in their molecular structure. Although these soft groups reduce the thermal stability of PEI to a certain extent, they inject excellent processing properties into PEI. For example, PEI can achieve hot-winding operations without relying on the adhesive layer [18,19,20].

PI insulation paint can be used as insulation impregnation paint for motor winding and insulation topcoats for wires in motors. It can also be used as an anti-corona paint for grooves and the ends of high-voltage large motors. At 250 °C, the service life of PI-enameled wire can reach more than 10,000 h. The mixed polyimide (PI) film has good viscosity controllability, and the heat-resistance grade can reach more than 240 °C. Sun Fei et al. developed a hybrid polyimide (PI) paint film with good viscosity controllability, a dielectric-loss steep-rise temperature of more than 250 °C, and a heat-resistance grade of more than 240 °C [21]. Xu Yong et al. prepared an impregnated insulating paint with low viscosity (2000~4000 mPa·s), low coating difficulty, and a high temperature resistance of up to 270 °C [22]. In order to improve the heat resistance of enameled wire, some studies have also used the sol-gel method to prepare silica (SiO_2_)-modified PI and PAI. Marikina et al. successfully developed a polyimide (PI) insulating paint containing SiO_2_ using tetraethoxysilane (TEOS) as a raw material. It was found that after thermal aging at 300 °C or 400 °C, the electrical breakdown strength of the silicon-containing hybrid film was significantly better than that of the PI film without SiO_2_, and the breakdown strength was increased several times over. After aging at 400 °C for 16 h, the hydrogen bonds formed by SiO_2_ nanoparticles and carbonyl groups in PI inhibited the thermal degradation process of the hybrid films to a certain extent, and reduced the size and number of voids formed by thermal degradation [23].Using trimellitic anhydride (TMA), 4,4‘-diaminodiphenylmethane diisocyanate (MDI), 4,4‘-diaminodiphenylmethane (DADPM), and TEOS, Morikawa et al. synthesized PAI/SiO_2_ hybrid films containing 33% SiO_2_. The glass-transition temperature of the films was increased to 270 °C [24]. However, the doping of SiO_2_ would lead to a decrease in the mechanical properties of the paint film and affect the application of the material. He Xiaodong et al. developed a thermoplastic PI cable material with good insulation performance, processing performance, heat resistance and mechanical properties, which is used in nuclear power and aviation fields [25]. Nanoparticles have a quantum size effect and a specific surface effect. Their appropriate addition can significantly improve the dielectric properties of composites. Therefore, the preparation of an inorganic nanoparticle–organic-polymer-composite insulation layer has become a hot topic in current research [26,27]. Japan’s MASKAZU et al. [28] prepared a high-temperature-resistant polymer-SiO_2_-composite insulating layer by cross-linking PAI (polyamide imide) and silica. Yang et al. [29] successfully prepared a nano-modified polymer by using nano-TiO_2_ modified PAI, and the life of the electromagnetic wire was increased by seven-fold. Although there has been a lot of research, the current polyimide (PI) polymers still fail to break through the threshold of 300 °C in terms of heat resistance. It is urgent to find new research ideas to enhance the heat resistance of polyimide (PI) polymer materials.

### 2.2. Polytetrafluoroethylene (PTFE) Polymer Material

**Figure 2:**
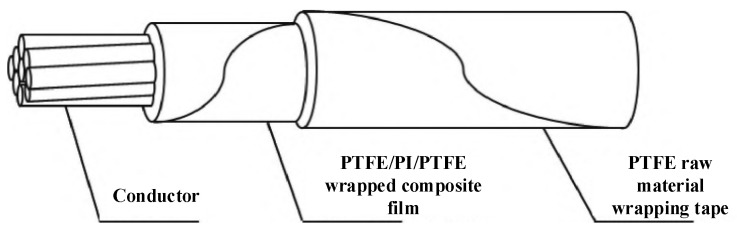
High-temperature-resistant composite PTFE film-wrapped aviation cable structure.

Polytetrafluoroethylene (PTFE) is a highly crystalline white thermoplastic known for its melting point of 327 °C. The molecular structure of PTFE is very symmetrical and has no branched chain. It is a linear polymer material and has no polarity between its molecules. In the crystalline state, PTFE presents a spiral structure and has a high-kneading property. At the same time, the main chain of fluorine-atom-covered-C-C bonds provides a protective shell for the carbon atom chain, which endows PTFE with excellent chemical resistance. Its strong carbon–fluorine bond also endows PTFE with an excellent high-temperature stability and an extremely high melting viscosity. PTFE can be used in a wide temperature range from −250 °C to 260 °C. It has an excellent chemical stability, electrical insulation, flame retardancy, and extremely low water absorption, and has the characteristics of being self-lubricating and non-sticky. It is widely used in switching equipment, especially as a key nozzle material in the circuit breaker arc extinguishing chamber, and its performance directly affects the breaking characteristics of the circuit breaker. In the aviation field, the use of cables is particularly critical, which requires excellent electrical insulation, high- and low-temperature resistance, thermal conductivity, flame retardancy, and wear resistance [30,31]. It is one of the mainstream manufacturing methods for high-temperature and high-thermal-conductivity aviation cables, to manufacture aviation cables using PI double-sided composite PTFE by wrapping–sintering technology. This technology takes full advantage of the high-temperature resistance of PTFE, through precision-winding and sintering processes, forming a strong and durable cable-protective layer, not only to ensure the reliability and stability of the cable at extreme temperatures, but also endowing good electrical insulation, as shown in Figure 2 [32,33].

### 2.3. Polyetheretherketone (PEEK) Polymer Materials

Polyetheretherketone (PEEK) is a semi-crystalline high-performance engineering plastic. As a member of the polyaryletherketone family, its chemical structure shows a high degree of order. This material has been highly valued for its high-temperature resistance, chemical-corrosion resistance, and oxidation resistance, and has become an ideal choice for high-performance composite matrix resins. PEEK has excellent strength and rigidity, excellent heat resistance, significant self-lubricating properties, fire resistance, stability of physical structure, and good electrical insulation. Compared with other high-temperature-resistant plastics, PEEK is one of the highest heat-resistance grades and has the best comprehensive properties in special engineering plastics, which can be used for a long time at 250 °C [34,35,36,37]. Although the softening phenomenon of PEEK above 200 °C limits its performance in high-temperature environments, the use temperature of PEEK can be improved by modifying the material, so as to improve its application performance at high temperatures [38]. Zhou et al. used SPEEK as a coupling agent to prepare PEEK/CaCO_3_ composites by melt-blending processes. The glass-transition temperature of the composites increased and the melting point decreased, which improved the use temperature of the materials and reduced the processing temperature [39]. Zhu Chengshuang prepared PEEK/CoPEEK/carbon black blends. They conducted a study on the heat resistance and vibration properties of CoPEEK and PEEK/carbon black blends. The results indicate that the hot deformation temperature of pure PEEK is 145 °C. However, when the mass ratio of PEEK/CoPEEK/carbon black is adjusted to 30/70/1, the hot deformation temperature of PEEK/CoPEEK/carbon black significantly increases to 265 °C, which is 120 °C higher than that of pure PEEK [40].

### 2.4. Silicone Rubber

Silicone rubber is a unique semi-organic and semi-inorganic polymer. Its molecular structure is composed of a Si-O-Si inorganic main chain and an organic side chain. This structure endows silicone rubber with both the flexibility of organic polymers and the high-temperature resistance of inorganic polymers. The high-temperature resistance of silicone rubber is affected by its molecular structure, moisture and impurity content, and environmental factors [41,42]. The high-temperature resistance of silicone rubber can be enhanced by several methods: one is to modify its molecular main chain and side chain; the second is to select the appropriate crosslinking agent to enhance the stability of the crosslinking bond; the third is to add high-temperature-resistant additives. Some other methods such as purification, the surface treatment of fillers, and the combination of rubber materials also help to improve its high-temperature resistance [43,44,45,46,47,48]. Lai Liangqing et al. found that phenyl ether silicone rubber had better air-heat-aging resistance than phenyl silicone rubber. The initial decomposition temperature and maximum decomposition temperature of phenyl ether silicone rubber were 461.67 °C and 648.76 °C, respectively, which were about 80 °C and 110 °C higher than those of phenyl silicone rubber [49]. Zhang et al. successfully synthesized vinyl-functionalized boron-containing hybrid silicone rubber by using triethoxyvinylsilane and boric acid as raw materials under solvent-free conditions. It had a better thermal stability than ordinary silicone rubber with only Si-O-Si bonds, and its residual mass fraction at 800 °C was about 82.4% [50,51]. Ge et al. synthesized a caged octapoly (dimethylsiloxy) silsesquioxane, which has a high Si-H bond reactivity and can undergo hydrosilylation reactions with the double bond in vinyl silicone rubber. The initial decomposition temperature of silicone rubber was increased by 54.43 °C when it was added as a crosslinking agent to vinyl silicone rubber [52].

The fundamental reason for limiting the tolerance temperature of organic insulation is the high-temperature degradation of polymer molecules. The tolerance temperature of the existing organic insulation is not more than 250 °C, which can only be increased by tens of degrees by using methods such as hybridization and branching, and the tolerance temperature is still not more than 300 °C. At present, the tolerance temperature of organic high-temperature-resistant electromagnetic wire insulation materials is not more than 285 °C, which makes it difficult to cope with the extremely high-temperature working environment above 400 °C, such as in the fields of aerospace and atomic energy.

## 3. Organic–Inorganic-Composite High-Temperature-Resistant Electromagnetic Wire

Organic–inorganic composite insulation materials have the advantages of high-temperature resistance and a high insulation of inorganic materials and high adhesion, strong crack resistance, and a good flexibility of organic materials [53,54]. Yang et al. [55] developed a new type of special high-temperature resistant coating. It has excellent high-temperature resistance, heat, and humidity resistance and insulation, and can be used at 500 °C. Liu et al. [56] used the artificial spraying method to prepare the paint film, which can withstand a high temperature of 600 °C at an applied voltage of 250 V. Wang [57] studied the performance of silicone coating and silicone–acrylic coating, finding that they could withstand temperatures of 500 to 600 °C. The coating improves the high-temperature resistance and corrosion resistance of the coating by the low-melting-point glass powder in the coating.

Due to the high price of pure silicone resin and the weak intermolecular force of the resin, the coating film has insufficient solvent resistance and substrate adhesion. In order to overcome these problems, people usually use other resins for modification. At present, the commonly used modified resins include acrylic resin, polyurethane, epoxy resin, and alkyd resin. Guo et al. [58] successfully prepared a hydroxyl-containing silicone resin by hydrolysis and the condensation of alkoxysilane monomers. A polyurethane-modified silicone resin high-temperature-resistant coating with room temperature curing and high-temperature resistance up to 700 °C can be prepared using this resin with a polyurethane curing agent and mica powder, aluminum powder, quartz powder, and other fillers after being treated at different temperatures for 1 h.

In addition, Feng et al. [59] successfully synthesized epoxy-modified silicone resin by using an alkoxysilane monomer and epoxy resin. Subsequently, toluene 2,4-diisocyanate was used as a coupling agent, diethylenetriamine was used as a curing agent, and fillers such as silica and titanium dioxide were added to prepare a high-temperature resistant coating that can withstand up to 450 °C. Lian Weizhen et al. [60] successfully prepared anti-corrosion and heat-insulating coatings with high-temperature resistance up to 500 °C by using an epoxy-modified silicone resin as the base material and using composite hollow glass microspheres and low-temperature molten glass powder as fillers. At the same time, they also discussed the influence of the proportion of hollow glass microspheres in the filler on the thermal conductivity of the coating. The results showed that when the proportion of hollow glass microspheres in the filler was 20~40%, the coating showed excellent heat resistance. Pang Y X et al. [61,62] conducted a detailed study of different organosilane compounds by designing and synthesizing a variety of nanocomposites. They finally selected a nanocomposite synthesized by MTMS, GPTMS, and PhTES to develop a ceramic/inorganic–organic nanocomposite. This new material not only has excellent thermal stability and mechanical properties, but also can remove its internal epoxy polymer network at high temperatures, thereby maintaining good insulation performance. Although this material can withstand a temperature of up to 500 °C, its organic composition will degrade rapidly at a high temperature of 550 °C, resulting in cracks and the shedding of the coating.

The short-term resistance temperature of some silicone resin coatings can reach more than 500 °C. By modifying the main chain and branch chain or doping glass and ceramic fillers, secondary film formation can be achieved to further improve its high-temperature resistance. At present, scholars have not yet studied its electrical-insulation properties in depth, and whether it is suitable as an insulating material for high-temperature-resistant electromagnetic wires remains to be discussed. However, the author believes that this is the future development direction of organic high-temperature-resistant insulating materials.

## 4. Inorganic Insulated High-Temperature-Resistant Electromagnetic Wire

The traditional polymer-based insulated high-temperature-resistant electromagnetic wire cannot operate at a high-temperature environment above 300 °C. Due to the excellent high-temperature stability of inorganic insulating materials, the temperature resistance of electromagnetic wires using them as insulating materials can reach more than 600 °C, far exceeding the temperature limit of organic polymer insulating materials. At present, the insulating materials of inorganic insulated high-temperature-resistant electromagnetic wire are metal oxides (Al_2_O_3_, MgO, SiO_2_), glass, glass fiber, and so on. The selection principle of the conductor material and the insulation material of inorganic insulation high-temperature-resistant electromagnetic wire is different from that of the existing traditional organic polymer insulation. Next, the selection of the conductor material of inorganic insulation high-temperature-resistant electromagnetic wire and the selection and preparation method of the inorganic insulation layer will be introduced in detail.

### 4.1. Inorganic Insulation Type for High-Temperature-Resistant Electromagnetic-Wire Conductor Material

When the temperature exceeds 200 °C or even higher, copper will be oxidized, which seriously affects its conductivity, ductility, and strength. Therefore, a single high-purity copper is no longer suitable as a conductor material for high-temperature electromagnetic wires. To prevent the oxidation of Cu at high temperatures, usually a thin sheath is applied by either ‘electroplating’ or ‘cladding’. Gu Chuanmeng et al. found that the conductivity of silver wire and aluminum wire is almost unchanged during the heating process, but the conductivity of nickel-plated copper wire will decrease slightly, and the decrease in copper wire is more obvious [63]. Although the conductivity of aluminum wire decreases slightly at high temperatures, the melting point of aluminum is 660 °C, and it will begin to soften above 400 °C, and the mechanical properties are greatly reduced. Silver wire shows good oxidation resistance at high temperatures, but its high cost limits its wide application. It is also an effective method for improving the performance of high-temperature electromagnetic-wire conductor materials by using some excellent metal compounds at high temperatures, such as Nb_3_Sn and MgB_2_, to coat copper wire. Covering metal compounds with excellent oxidation resistance can prevent the diffusion of copper at high temperatures and ensure the insulation performance of electromagnetic wire at high temperatures [64,65]. Cr is also a material with excellent oxidation resistance at high temperatures, but the manufacturing cost of chromium coating is too expensive [66]. Considering various factors, nickel-plated copper wire is a more suitable conductor material.

Another potential problem of high-temperature-resistant conductor materials is the diffusion of Cu atoms to the protective layer at high temperatures, which leads to a decrease in the resistivity of the insulating layer at high temperatures and the deterioration of the insulation. Zijing Wang [67,68] shows that a typical AWG20-Class3 Ni-coated Cu wire continuously annealed at 400 °C for 5500 h and the resistivity increased by 6.9%, and promoted a concentric circle model to simulate changes in composition and effective resistivity in the Ni–Cu wire as a function of time. He noted that a good agreement between the simulated and experimental data for effective resistivity was only achieved by employing effective diffusion coefficients corrected for microstructural effects.

In summary, nickel-plated copper wire is the preferred conductor material for high-temperature electromagnetic wire at present, but the conductivity decrease and mechanical aging caused by Cu atom diffusion at high temperatures are still worthy of further study.

### 4.2. Preparation Method of Inorganic Insulated High-Temperature-Resistant Electromagnetic Wire

The construction of the inorganic insulation layer on the electromagnetic-wire conductor material is part of the process of preparing the inorganic insulation layer on the metal surface. At present, the methods for preparing inorganic insulating layers on metal surfaces mainly include thermal spraying, vapor deposition technology (including physical vapor deposition and chemical vapor deposition), plasma electrolytic oxidation, the sol-gel method, and the slurry sintering method. The author believes that due to the small size and low heat capacity of the substrate material of the electromagnetic wire, when the ceramic coating is prepared by thermal spraying on the surface of the wire, the thermal stress of the coating and the substrate will be large due to the fact that the heat cannot be dissipated in time; the substrate will be deformed, and the coating will crack or even fall off. At the same time, the price of thermal spraying is expensive, so there is no suitable spraying method. For vapor-deposition technology, its deposition rate is low, and it also faces the process problem of there being no deposition device for electromagnetic-wire conductor materials. The three methods of plasma electrolytic oxidation, the sol-gel method, and the slurry sintering method can use the roll-to-roll method of industrially produced wires to construct an inorganic insulating film on the electromagnetic wire substrate, which is a common method for preparing high-temperature-resistant inorganic insulating electromagnetic wires. The focus of these methods is on the selection of inorganic insulating materials and the design of slurry systems. For the selection of ceramic materials, the following four requirements should be met [69]:

- A low dielectric constant;
- A high dielectric strength;
- A high thermal conductivity;
- A coefficient of thermal expansion matching the matrix.

Electrically insulating ceramics that meet the requirements mentioned above include Al2O3, BeO, AlN, and some types of glass. These ceramics exhibit low relative dielectric constants (<15), sufficient dielectric strength (>15 V/μm), high thermal conductivity (>20 W/m^−1^), and thermal-expansion coefficients similar to that of nickel and copper [70].

#### 4.2.1. Coating Sintering Method

**Figure 3:**
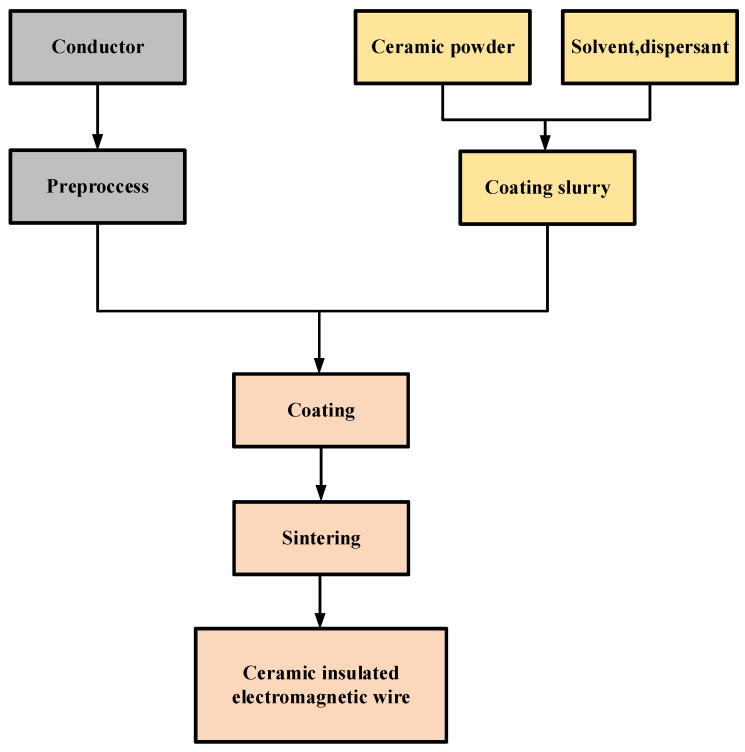
Basic steps of coating sintering method.

The coating sintering method is to prepare the ceramic slurry as the insulating layer into a ceramic slurry, and the slurry is then coated onto the wire by dipping and pulling, and then the slurry and the wire are co-fired into an inorganic insulated electromagnetic wire. The basic steps are shown in Figure 3.

During the dip-coating process, the effective contact between the substrate and the sol is very important, and the key lies in the wetting ability of the sol to the substrate. The degree of wetting can be evaluated by measuring the contact angle; that is, the angle formed between the droplet on the solid surface and the solid–liquid interface. This angle, usually expressed as θe, is an indicator for evaluating the wettability of liquids on solid surfaces. The size of the contact angle directly reflects the infiltration of the liquid on the solid surface: a contact angle close to 0° indicates superhydrophilicity, indicating complete wetting; a contact angle less than 90° indicates hydrophilicity, that is, the partial wetting state; a contact angle equal to 90° is the boundary between wetting and non-wetting; a contact angle greater than 90° indicates hydrophobicity; and that greater than 150° indicates superhydrophobicity, where the liquid hardly wets the solid surface.

Although most of the electrical insulating ceramics are basically composite candidates for high-temperature-electromagnetic-wire insulation materials, the densification sintering temperature of these electrical insulating ceramics is above 1500 °C, and they cannot be co-sintered with the matrix copper below the melting point of copper (1081 °C). The requirement of the coating sintering method for candidate inorganic insulating materials is to have an inherent sintering densification temperature lower than the melting point of the matrix and sufficient dielectric strength. The successful development of such materials would indicate an alternative route to solve the problem of insulation materials for use in high-temperature electrical wires.

The selection of slurry formula is basically consistent with the slurry formula of the tape-casting process in the field of ceramic molding, except for inorganic powder, including the solvent, dispersant, binder, plasticizer, and other additives. The slurry system includes an organic system and inorganic system. The commonly used solvents for organic systems are toluene, xylene, ethanol, etc. In actual production, binary azeotropic solvents such as ethanol/toluene and ethanol/trichloroethylene are commonly used [71]. Because the organic solvent has the characteristics of compatibility, easy volatilization, low evaporation latent heat, and low surface tension, and can prevent the hydration of ceramic powder, the organic tape-casting system has many advantages, such as a wide selection range of additives, a fast volatilization of the solvent, a short drying time, and so on. It is easy to obtain a ceramic membrane with a uniform structure, small defect size, high strength, and good flexibility. However, organic solvents have a certain toxicity, which inevitably brings harm to human beings and the ecological environment. In addition, the production cost is high, the organic content of the finished product is high, the density is low, and the glue-discharge process is easy to perform. All these restrict the development of organic ceramic slurry [72,73]. The water-based casting system overcomes the disadvantages of the organic casting system, such as harm to the environment and the low density of finished products, and is suitable for large-scale production. However, there are the following main problems in the water-based tape-casting system [74,75]: (1) a low solvent-evaporation rate; (2) when the binder content is high, the shrinkage rate of the green body is large; (3) due to the presence of hydrogen bonds, the powder is agglomerated; (4) it is sensitive to the change in process parameters, so the quality of the green film is low; and (5) the green body is brittle and easy to crack during drying. At present, in the field of high-temperature electromagnetic wire, because the dielectric properties of the insulating layer are greatly affected by the defects of the ceramic film, the organic tape-casting system is still used for sintering preparation.

Rao et al. [76] used modified-lead glass powder, talcum powder, and montmorillonite as solutes and butyl titanate and xylene as solvents to prepare a high-viscosity ceramic slurry. Pb-Si-Mg-Ti-O ceramic coatings were rapidly prepared on a nickel surface by dip-coating and low-temperature sintering. The coating had a good insulation at 350 °C and good flexibility with the deformation of the nickel sheet. The ceramic coatings prepared by pulling were relatively flat, which indicated that the glass phase could bond the surrounding ceramic particles and fill the tiny cracks during the rapid sintering process. The uniformity and integrity of the coating increased with the increase in solid content. However, when the solid content is too high, the thickening of the slurry will lead to an increase in coating thickness and porosity, which will affect the performance of the coating.

Kreidler et al. [77] fabricated the signal line for an aero-engine sensor by sintering alumina ceramic on the surface of platinum wire at 1500 °C using an organic tape-casting slurry formula with solvents of ethanol and toluene. Jun Lu et al. [78] developed a ceramic insulating coating on Bi2Sr2CaCu2O8-x circular wires for high-field magnet applications. The coating was composed of TiO2 nanopowders, polymer binders, and other additives. The conductor was impregnated in the ceramic slurry by the pull-film method, and the thickness could be controlled between 10 and 30 μm after sintering. Zijing Wang [79,80] reported a new LTCC material based on the 11ZnO-10MoO_3_ formula, which can be sintered at 850 °C and has good dielectric and thermal properties, making it an ideal choice for dip-coating and co-firing applications. They further coated this material on a nickel-plated copper conductor wire by the impregnation method, and formed a ceramic film with a thickness of 47.3 μm and a dielectric strength of 24.2 kV·mm^−1^ on the Ni test piece.

The formula of the ceramic slurry used in the coating sintering method is relatively well developed. A slurry formula with a good wettability with the conductor should be selected, and the discharge temperature should be less than the sintering temperature. For the selection and design of inorganic insulation materials, first of all, the insulation material should not contain alkali metals, including Na, Ka, Li, etc., because the alkali metal ion conductivity is high at high temperatures, which is extremely unfavorable for the insulation. Secondly, components that can be co-sintered with the substrate should be designed, and the lower the sintering densification temperature, the better. Finally, high electrical insulation performance, low relative dielectric constants, and thermal expansion coefficients matching the substrate can be achieved by increasing the second phase. The author believes that in the design of insulating materials, ideas should be sought by referring to the LTCC field, such as CaO-B_2_O_3_-SiO_2_, Al_2_O_3_-B_2_O_3_-SiO_2_, BaO-SrO-SiO_2_, etc., whose co-sintering densification temperatures are lower than 900 °C. After adding low-melting-point glass to the above system, the sintering temperature can be further reduced and the density can be improved. At the same time, other metal oxides are introduced to regulate the thermal-expansion coefficient and reduce the sintering temperature.

#### 4.2.2. Sol-Gel Method

**Figure 4:**
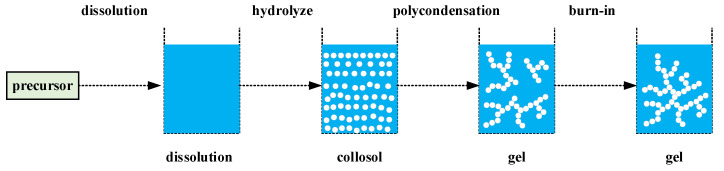
Process flow of glue-gel method.

The sol-gel method is a method of preparing thin films and coatings. This technology uses highly active compounds as precursors to mix with raw materials in a liquid state and undergo hydrolysis and condensation reactions to form a stable sol system. This sol gradually polymerizes during aging and transforms into a gel with a three-dimensional network structure, in which the solvent loses fluidity. After that, the gel is solidified into the final product through the drying and sintering processes, and the technical process is shown in Figure 4. The dip-coating experiment is performed to immerse the substrate into the sol fluid. When the fluid wets the substrate, the substrate is pulled out vertically and slowly at a certain speed. The sol adheres to the surface of the substrate under the action of viscous force, surface tension, gravity, etc., forming a liquid film. The thickness of the liquid film and the morphology of the formed free surface are affected by the fluid properties, the surface properties of the substrate, and the parameters of the dip-coating process. Celik et al. [65] demonstrated that employing MgO/ZrO_2_ as an insulating layer on silver and copper-coated Nb3Sn wires can result in a higher breakdown voltage and a lower dielectric constant.

The sol-gel method can be used to coat the sol on the conductor by dipping and pulling. Li [81] applied a coating composed of sodium silicate, oxalic acid, and aluminum dihydrogen phosphate on the surface of electrical steel using the dip-coating method, and successfully prepared a high-temperature-resistant insulation coating. This coating works stably at 300 °C and can withstand a temperature of up to 600 °C. During the study, it was found that the addition of sodium silicate can promote the dehydration condensation reaction of AlH_3_ (PO_4_) _2_ in the coating at 200 °C, and generate AlH_2_P_3_O_10_. This substance can form a chemical bond with the surface of electrical steel in an acidic environment, that is, MeAlP_3_O_10_, thereby improving the adhesion of the coating and the dispersion of the coating. At the same time, the addition of a chelating agent can maintain the stability of the coating. Wang et al. [82] successfully prepared TiO_2_ thin films on glass substrates by the sol-gel method and dip-coating technique, using tetrabutyl titanate and ethanol as solvents, and systematically studied the relationship between dip-coating times and dip-coating speeds. It was found that a smooth and dense TiO_2_ film can be obtained by dip-coating twice and controlling the dip-coating speed at 3 to 7 cm per minute. When nano-silicon-based materials are used as insulation coatings, cracks may occur in the coating due to volume shrinkage caused by heating. In order to solve this problem, the introduction of non-shrinkage materials to prepare composite coatings is an effective strategy. In this composite coating, nanomaterials serve as the matrix phase to provide adhesion and the necessary mechanical properties, while granular ceramics serve as the dispersed phase to reduce shrinkage during heat treatment and enhance the dielectric strength of the coating. Zhao X et al. [83] prepared a high-temperature electrical insulating coating by depositing Al_2_O_3_ on the surface of a NiCrAlY coating using the sol-gel method. This composite coating has a resistance of more than 1 MΩ in the range of 600 °C to 800 °C, and after thermal shock and thermal fatigue tests, no cracking or peeling was found, thus solving the electrical-insulation problem of nickel-based superalloy substrates.

The inherent cracking problem of the sol-gel method in its drying stage limits the application of this method in the field of electromagnetic wires, which are extremely sensitive to coating defects. Moreover, the coating composition of the sol-gel is often relatively simple, and it is difficult to use a single metal oxide to match the insulation and mechanical properties of high-temperature-resistant electromagnetic wires at the same time. The author believes that the sol-gel method should not be used in the field of high-temperature-resistant electromagnetic wire.

#### 4.2.3. Plasma Electrolytic Oxidation

**Figure 5:**
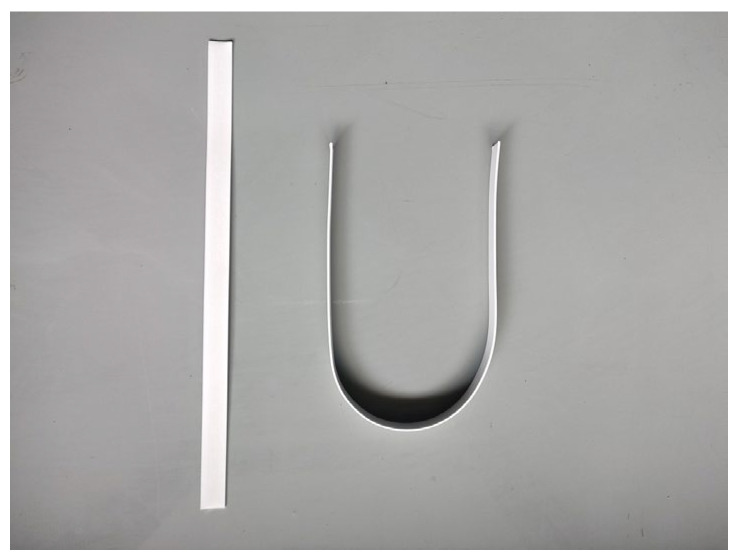
Plasma electrolytic oxidation of high-temperature-resistant ceramic electromagnetic wire.

**Figure 6:**
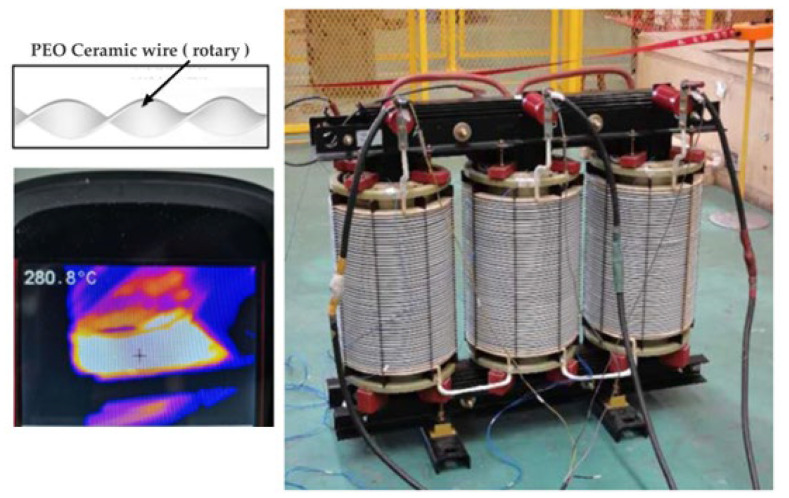
Dry-type transformer prototype.

Plasma electrolytic oxidation (PEO) is an efficient and environmentally friendly advanced surface-treatment technology, which is mainly used in valve metals such as aluminum, magnesium, titanium, zirconium, niobium, tantalum, and their alloys. The method is performed by immersing the metal material in an alkaline electrolyte and applying a high voltage to initiate the surface-breakdown micro-arc discharge. The local high-temperature and high-pressure environment generated during the discharge process promotes the in situ formation of functional ceramic coatings on the metal surface with metal oxides acting as the main body and elements in the electrolyte participating in doping or mixed modification [84]. At present, the research on the insulation characteristics of micro-arc oxidation ceramic insulation film mainly focuses on the influence of process parameters on the insulation performance of ceramic film. Zhang Guoyuan [85] and He Xiang et al. [86] studied the influence of power parameters on the insulation properties of micro-arc oxidation ceramic films. The results show that the volume resistivity, insulation resistance, and breakdown voltage of ceramic films increase with the increase in positive and negative pulse duty cycle and frequency. Gutsalenko et al. [87] measured the volume resistivity and breakdown voltage of micro-arc oxidation ceramic films under different electrolyte ratios. When the electrolyte was 1 g/L potassium hydroxide and 6 g/L sodium silicate, the volume resistivity of the ceramic film was 8.9 × 10^9^ Ω·m, and the breakdown strength was 13.8 V/μm. On the other hand, Qu et al. [88] studied the variation in the dielectric properties of zirconium alloy micro-arc oxidation film using temperature. In the range of −100~250 °C, the dielectric constant, dielectric loss, and conductivity of the ceramic film in the low-frequency region increased with the increase in temperature, and the temperature had little effect on the high-frequency region. The author’s team studied the law of the micro-arc oxidation discharge mode on an aluminum substrate, and prepared a high-temperature-resistant micro-arc oxidation electromagnetic wire, as shown in Figure 5. The author studied the power frequency breakdown characteristics of the electromagnetic wire [8,89] and applied it to a single-phase switched reluctance motor, which increased the stable operating temperature of the motor to 350 °C [69]. At the same time, the author’s team also developed a dry-type transformer based on an aluminum-based plasma electrolytic oxidation high-temperature electromagnetic wire. As shown in Figure 6, under the rated load condition, the hottest spot’s temperature rise for this 10 kV ceramic-insulated dry-type transformer was 121.98 K, which meets the requirements of H-level insulation. It can withstand the high temperature of 350 °C under short-term thermal-overload conditions. The loading started from a 50% overload, and the test was continued for 30 days. After loading to 200% of the rated current, the winding temperature was maintained at about 280 °C for a significant duration for 14 days, and the winding insulation was not damaged. The total weight value was reduced by 18.96% compared with that before optimization [90].

Although, the micro-arc oxidation technology can in situ grow a layer of alumina ceramic film on the aluminum conductor, and the bonding strength between the film and the substrate is much higher than that of the electromagnetic wire prepared by the slurry method, sol-gel method, and other methods. However, the melting point and softening temperature of aluminum itself are low. When the temperature exceeds 400 °C, the mechanical properties of the wire are greatly reduced. Using the high-temperature electromagnetic wire of the micro-arc oxidation ceramic film, it may be difficult to withstand the high-temperature environment of long-term temperatures exceeding 400 °C.

The technical route and performance comparison of inorganic insulated high-temperature-resistant electromagnetic wire are listed in Table 1.

### 4.3. Inorganic High-Temperature-Resistant Electromagnetic Wire Products

**Figure 7:**
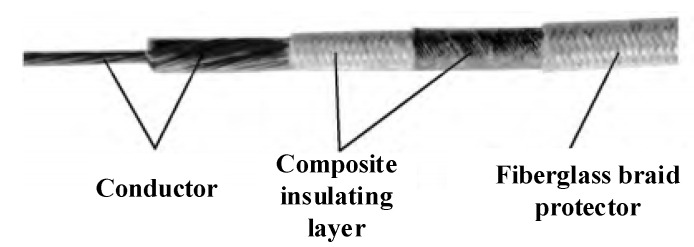
Double-insulated 500 °C-resistant cable of Far East Cable Co. LTD.

**Figure 8:**
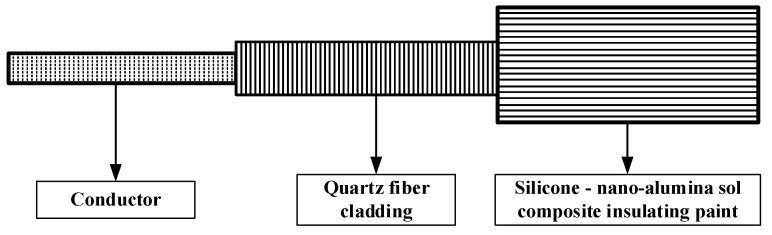
Nano-silicone alumina sol-coated quartz-fiber-wrapped wire structure.

The existing developed products of electromagnetic wire with a long-term resistance to high temperatures above 400 °C are all inorganic insulated electromagnetic wire. Far East Cable Co. LTD (Yixing, China) uses a nickel-plated copper wire as a conductor, and the insulation adopts inorganic glass fiber weaving and inorganic high-temperature refractory insulation to synthesize a mica tape-wrapping composite structure. An electromagnetic wire that can withstand a 2500 V power frequency voltage at 500 °C was prepared, as shown in Figure 7. The 23rd Research Institute of China Electronics Technology Group has developed a nickel-plated copper wire as a conductor, and the insulation layer adopts a high-silica glass fiber yarn-wrapping and high-temperature coating-liquid impregnation structure. The bending radius of the electromagnetic wire is less than 5D, which can withstand high temperatures of 500 °C for a long time. Tuowen Company (Zhuhai, China) has developed a high-temperature-resistant electromagnetic wire with SiO_2_ as the insulating layer. The bending radius is less than 7D and can withstand a 1000 V DC voltage. The electromagnetic wire of the Pozh series in Russia is wound with quartz fiber and silicate aluminum magnesium fiber, and is impregnated with organic silicate. It can run continuously for 1000 h at 500 degrees Celsius, and the power frequency breakdown voltage is 480 V. American Ceramawire company (Elizabeth City, NC, USA) has developed two kinds of high-temperature-resistant electromagnetic wires with conductor materials of 27% nickel-coated copper and all-nickel. The insulating layer is a glass coating, which has a similar thermal expansion coefficient to the matrix of nickel, and has a high-radiation resistance. The high-temperature electromagnetic wire developed by the Illinois Institute of Technology in the United States uses a high-temperature-resistant inorganic ceramic coating as an insulation layer and nickel plating on the surface of a modified copper alloy as the core wire. The operating temperature can reach 1000 °C. The THERMO—COAX company in France uses a new type of high-temperature-insulation adhesive as the insulation layer of electromagnetic wires. After high-temperature sintering, the resistivity of the insulation layer reaches 10 MΩ·m at 600 °C, which has been successfully applied in aviation and aerospace fields. The Shanghai Cable Research Institute Co., Ltd. [91] prepared a high-temperature-resistant insulating layer by coating the quartz-fiber-wrapping layer with nano-silicone alumina sol through organic–inorganic compounding technology. The resistivity of the insulating layer was greater than 1 MΩ·m at 800 °C, and the breakdown voltage at room temperature exceeded 2000 V, as shown in Figure 8.

## 5. Discussion

At present, conventional electromagnetic wires use polymers as insulating materials. High-temperature-resistant polymer insulation material is the development direction of high-temperature-resistant electromagnetic wire. Polymer dielectric materials with temperature resistances exceeding grade H have been rapidly developed in recent years, such as polyimide, polytetrafluoroethylene, and polyether ether ketone. In terms of structural characteristics, these high-temperature-resistant polymer materials often have strong conjugate interactions between molecular structures or chemical bonds such as hydrogen bonds, which are easily formed within the molecular structure, resulting in strong interaction forces within and between molecular chains, which are directly manifested as excellent high-temperature resistance (high glass-transition temperature, high thermal-decomposition temperature, excellent high-temperature dimensional stability, etc.), excellent dielectric properties (high insulation strength, high volume and surface resistivity, etc.), excellent environmental stability (chemical environment, atmospheric environment, space environment, etc.), and good mechanical properties. However, the temperature tolerance of these high-temperature-resistant polymer materials does not exceed 280 °C.

The use of inorganic particles to modify polymers to form composite materials/nanocomposites has long been used to improve the thermal properties of polymers, such as TiO_2_, SiO_2_, Al_2_O_3_, and ZnO. It is beneficial to disperse the space charge in the material, reduce or eliminate the stress caused by the uneven local electric field, weaken the partial discharge intensity, and improve the thermal conductivity of the insulating material. It can increase the temperature of the insulating material able to be withstood by more than 10 °C, but it still cannot exceed 300 °C.

When the electromagnetic wire must work in an environment above 300 °C, in order to avoid the conductivity reduction caused by the oxidation of the conductor material at high temperatures and the insulation deterioration caused by the diffusion of the conductor copper atoms into the insulation layer, a protective layer of Ni is generally coated on the Cu conductor, which is the consensus and common practice in the field of high-temperature electromagnetic wire. For insulating materials, organic–inorganic-composite insulating materials or pure inorganic insulating materials must be used.

Different from polymer organic insulating materials, inorganic–organic-composite insulation materials are a class of composites in which the inorganic phase and the organic phase are mixed at the molecular level and connected by covalent bonds. Most of them are derived from sol-gel chemistry, using functional organic metal alkoxides as precursors, usually organically, rather than being prepared by traditional blending processes. A variety of organic and inorganic components can regulate the specific properties of the resulting material. More interestingly, due to the finer dispersion of the organic and inorganic phases, synergistic effects can be achieved. However, at a temperature higher than 300 °C or even higher, the thermal effect causes the main chain of the silicone molecule to continuously generate new cyclosiloxane small molecules through random rearrangements, resulting in a continuous decrease in the molecular weight of the remaining silicone-gel molecular system. The cyclosiloxane molecules will lose a stable ring structure in the later stage of the pyrolysis of the silicone gel, and even continue to crack, resulting in the gradual disintegration of the entire silicone-gel molecular system. The addition of some ceramic particles such as Al_2_O_3_, SiO_2_, and TiO_2_ can reduce the decomposition rate of organic silicone and increase the initial decomposition temperature. The addition of some glass fillers can make the organic–inorganic composite insulation material form a secondary film at high temperatures and improve the heat-resistance level. However, organic–inorganic-composite insulation materials still cannot avoid the process of molecular chain cracking at high temperatures, and defects such as bubble cracks will also appear at high temperatures, which are potential insulation risks for high-temperature-resistant electromagnetic wires. The organic–inorganic-composite insulated high-temperature-resistant electromagnetic wire is only suitable for short-term high-temperature operation, and it is difficult to operate under long-term high-temperature conditions.

Inorganic insulation materials can avoid the problem of insulation degradation caused by the cracking of insulation materials at high temperatures, and can operate stably for a long time at 500 °C and above. At present, the preparation process of inorganic insulation high-temperature-resistant electromagnetic wire is roughly divided into four kinds, the slurry-coating sintering method, the sol-gel method, the plasma electrolytic oxidation method, and the fiber-winding method. The coating sintering method is similar to the sol-gel method. The slurry or sol is first configured, and is then impregnated with the surface of the electromagnetic wire through the roll-to-roll process, and then sintered at a high temperature to form an inorganic insulation layer. In the selection of ceramic materials, on the one hand, the appropriate initial melting temperature and desired dielectric properties should be considered; on the other hand, the physical and chemical properties of the coating and the substrate should be considered. In terms of dielectric properties, an insulating layer with a high breakdown voltage should be selected, so semiconductors or components with poor insulation should not be added. At the same time, the coating and the conductor material should have the same or similar expansion coefficient. If the thermal-expansion coefficient of the conductor is greater than the thermal-expansion coefficient of the coating, the shrinkage of the conductor will be larger than that of the coating during cooling, and stress will be generated between the conductor and the coating, so that the coating will be subjected to compressive stress. The coating retains moderate compressive stress, which is beneficial to the quality of the coating. When the compressive stress exceeds a certain limit, it will cause the coating to peel off. Similarly, if the thermal-expansion coefficient of the conductor is less than the thermal-expansion coefficient of the coating, the shrinkage of the coating will be greater than that of the conductor during cooling, and the coating will be subjected to tensile stress. Once the limit is reached, the coating will crack. The coating prepared by these two methods is physically bonded to the substrate, and the bonding force is poor. In the sintering process, due to the removal of organic components, insulation defects such as pores and cracks will inevitably occur. In terms of the time cost of the process, both of the two technical routes include the process of ceramic sintering, so the production time is at the hour level and is slow.

The goal of preparing inorganic high-temperature-resistant electromagnetic wire by plasma electrolytic oxidation is to immerse the conductor in the electrolyte, change the line by the traction device to react, and complete the growth of the inorganic insulating layer in more than ten minutes. Therefore, the time cost of its production is superior to the first two technical routes. However, the current plasma electrolytic oxidation can only be carried out on the valve metal. For electrical conductors, only Al can be used as the conductor material of electromagnetic wire. On the one hand, the melting point of aluminum is 660 °C, which begins to soften when it exceeds 300 °C, and the mechanical properties of aluminum wire begin to deteriorate. On the other hand, the conductivity of aluminum itself is lower than that of copper, and aluminum is often selected as the conductor material of electromagnetic wire depending on the weight. Therefore, the high-temperature-resistant electromagnetic wire prepared by the plasma electrolytic oxidation method is limited by the matrix material, and its working temperature generally does not exceed 350 °C. The high-temperature-resistant electromagnetic wire was prepared by wrapping the glass fiber. Firstly, the alkali-free inorganic glass fiber was woven and wrapped on the electromagnetic wire, and the conductor was generally nickel-plated copper wire. Then, it was impregnated with high-temperature-resistant insulating paint such as modified high-temperature-resistant silicone resin, alumina sol, and other inorganic insulating paint at high temperatures to fill the gap between fibers and improve the electric-field uniformity and dielectric insulation level of the insulating layer. The preparation method of this kind of electromagnetic wire is complicated, and the time cost of production is also hourly, but its dielectric insulation strength is excellent, and its toughness is higher than that of pure inorganic insulation film high-temperature-resistant electromagnetic wire. The comparison of high-temperature-resistant electromagnetic wire insulation materials are shown in Table 2.

## 6. Conclusions

High-temperature-resistant electromagnetic wire is one of the key core components of electrical equipment in aerospace, atomic energy, underground oil and gas, and other fields. In these extreme environmental fields, it is often required that the electromagnetic wire can work in high-temperature environments above 500 °C. Therefore, the research and development of high-temperature-resistant electromagnetic wire has important significance and urgent practical need.

At present, common organic insulating materials such as polyimide, polytetrafluoroethylene, polyether ether ketone, and so on can not tolerate high temperatures above 280 °C. Even if the group is modified and the inorganic filler is added, the tolerance temperature is no more than 300 °C. Although organic–inorganic-composite insulation can increase the tolerable temperature to more than 500 °C, in the high-temperature environments, with the decomposition of organic components, the film layer will produce certain pores and cracks, and may even lead to the peeling of the film layer, which also affects the secondary-film formation process. The temperature resistance of inorganic high-temperature-resistant electromagnetic wire is higher than that of organic–inorganic composites, and the film will not change in nature at high temperatures.

The inorganic insulated electromagnetic wire prepared by plasma electrolytic oxidation involves a metallurgical bonding between the film and the substrate, and the bonding is tight and not easy to break. The plasma electrolytic oxidation method is limited by the selection of conductor materials, and it is currently impossible to coat on copper wires and nickel-coated copper wires. The preparation of inorganic insulated electromagnetic wire by the slurry-coating sintering method and the sol-gel method is not limited by the type of conductor material, but there are some shortcomings in the preparation process, such as the poor wettability of slurry and sol to the substrate, and the poor adhesion between the coating and the substrate after film formation. At the same time, the inorganic insulating electromagnetic wire has the common disadvantage that the toughness of the inorganic insulating layer struggles to meet the inherent winding property of the electromagnetic wire.

Based on the research results and existing problems of high-temperature-resistant electromagnetic wire nationally and abroad, the author believes that for organic–inorganic high-temperature-resistant electromagnetic wire, the collaborative design of the inorganic filler, main chain, and side chain of an organic silicone resin should be studied in the future to ensure the insulation performance at high temperatures. For inorganic high-temperature-resistant electromagnetic wire, the toughening of the inorganic insulating layer should be studied in the future to ensure the winding ability of the electromagnetic wire. How to prepare a ceramic insulated electromagnetic wire with good winding properties, a high breakdown voltage, and a stable high-temperature performance will still be a difficult problem to overcome.

## Figures and Tables

**Table 1:** Technical route and performance comparison of inorganic insulated high-temperature-resistant electromagnetic wire.

Technical Route	Substrate	Bond Strength	Coating Quality	Dielectric Insulation Performance	Winding Performance	Temperature-Tolerance Level	Reference
Coating sintering	No restriction	bad	fair	fair	Bad	≥500 °C	[71,72,73,74,75,76,77,78,79,80]
Sol-gel	No restriction	bad	fair	fair	bad	≥500 °C	[81,82,83]
Plasma electrolytic oxidation	Restricted to valve metals, such as Al	good	fair	fair	fair	≤350 °C	[89,90]
Fiber wrapping	No restriction	fair	fair	fair	bad	≥500 °C	[91]

**Table 2:** Comparison of high-temperature-resistant electromagnetic wire insulation materials.

Types of Electrical-Insulation Materials	Electrical-Insulation Materials	Working Temperature	Merit	Demerit	Application
Organic type	Polyimide polytetrafluoroethylene polyether ether ketone	<=280 °C	Good winding property High dielectric insulation strength	Low temperature-resistance level	Conventional enameled wires and capacitor cables
Organic–inorganic type	organosilicon + inorganic filler	300 °C~500 °C	Taking into account the winding and high-temperature-resistance characteristics	Difficult to withstand high-temperature environments for long periods	Aerospace Oil downhole Nuclear
Inorganic type	Al_2_O_3_,SiO_2_, MgO ZnO-MoO_3_ PbO-MgO-SiO_2_ Na_2_O-B_2_O_3_-SiO_2_ K_2_O-PbO-SiO_2_	>=500 °C	High temperature-resistance level	Poor winding property	Aerospace Oil downhole Nuclear
